# Our Bureau of Information

**Published:** 1912-01-13

**Authors:** 


### OUR BUREAU OF INFORMATION.

### Will Matrons and Sisters Send Cases?

### Cases and Helpers.

The more civilisation has advanced, and the greater the prosperity of a people, the more difficult it becomes to provide the maximum of protection and comfort to dependents who, as products of human development, through no fault of their own, are ill-equipped for the service of life. It is a remarkable testimony to the goodness of human nature that one or more generous souls have been awakened to the cruel necessities and disablements under which each of very many members of the race labour, often from birth, and sometimes in later years from surroundings, absence of care or adequate treatment, or through inability to endure the strain entailed by the struggle for existence. It has been our privilege to examine closely into most if not all the methods at present available for the alleviation of human suffering, and of the various forms of disablement which lead to dependence, which militate against the power of maintenance. The conviction we have formed is that the means already available are so diverse and manifold, that if once we organise through The Hospital a system whereby the precise necessities of each dependant can be made plain, and the friendly co-operation of individual workers and co-operators is secured, almost if not quite all the needs might be readily supplied with a promptness which would in the aggregate represent a saving in human misery in these islands well-nigh impossible to exaggerate.

If we could spread this knowledge broadcast, and so awaken individual interest as to get every healthy wholesome mind to believe what we have just written, the roll of our Brotherhood of Service would soon contain the names of thousands of people all over the country. We propose in the next few issues of The Hospital to bring out and deal with individual cases of hardship and of individuals suffering from disablement which make them dependents. In this way we may place all healthy and intelligent people in the position of the Good Samaritan, if they care to follow his example. We offer a centre, a sort of living exchange, in which all who need, all who are willing to serve, and all who, though unable to serve, are willing to supply the means where money can help, may join hands in good fellowship. Thus wisely, prudently, self-denyingly and happily many may help themselves by helping others less fortunately placed who, without their aid, must endure suffering and misery, while by its means such evils may be prevented.

Few people have a wider opportunity than hospital sisters and district nurses to gain an intimate knowledge of the patient endurance by individuals of much suffering from circumstances which could be lightened by the co-operation of a brotherhood such as ours. We desire to interest the sisters and matrons of our public institutions, district nurses, and visitors in the work of the Brotherhood. We ask them to send us tersely written facts concerning any case which may come under their notice where help of an unusual or difficult kind, which they cannot otherwise obtain, may be needed. Sisters and matrons, district visitors and district nurses, clergy and ministers, and most hospital and institutional workers can do immense good by a little personal service in setting down the requirements of particular cases of which they have knowledge, of the type here described, and sending them to the Editor. This will enable us to deal with these cases, and at the same time to illustrate practically the force and truth of most if not all of what we have here urged. Is it too much to ask the fellow workers who have been associated with us in loving labour for the sick for so many years to send at any rate one such case NOW, while they have the matter in mind? We hope further that many if not all such workers will join the Brotherhood, because their co-operation is essential to the accomplishment of the maximum of good which it is capable of rendering to those members of the race who are least able to help themselves.

The following is a case in point:—

### Earning a Livelihood while Undergoing a Cure.

"Oxymoron" writes: Could you or any of your readers kindly tell me what chance, if any, there is for a young man of twenty, with incipient phthisis, to earn his living while leading a sanatorium life? He has had a good business training and is fond of farm pursuits. Do not some sanatorium superintendents take men like this as secretaries, or in some capacity connected with the institution?

### How to Make the Bureau a Success.

Everyone who uses or wishes to help our Bureau of Information must be kind enough to observe the following rules:—

(1) Postal replies cannot be given. Exception will only be made in very special cases at the Editor's discretion.

(2) Queries or answers relating to different cases must be written, or preferably typed, on separate sheets of paper, and the writing must never be crossed.

(3) Questions and replies received not later than Wednesday will, as far as possible, be dealt with in our issue of the following Saturday week.

(4) Letters must have attached the name and address of the sender, with a pseudonym for publication if preferred.

(5) All applicants for insertion in our list of Approved Homes must send a registration fee of five shillings. The omission of this leads to delay in the insertion of names.

(6) *All communications* for this department *must be accompanied by the coupon* to be found at the bottom of the third page of the cover on the inside, and should be addressed to the Editor of the Bureau of Information, c/o The Hospital, 29 Southampton Street, Strand, London, W.C.

Notice.—We invite workers of special experience and knowledge to offer help in replying to questions relating to their special department.

### Our List of Approved Homes.

We are pleased to add the following to our list of Approved Homes:—

Ivy House, Pidley, Huntingdon. Principal, Miss F. M. Moles. Children, sick, ailing, backward, and slight mental cases. Large garden, open-air school in summer. Situation, 199 feet above sea-level.

Ifield Lodge, Uckfield, Sussex. High situation with extensive views. Principal, daughter and sister of clergymen. Principal wishes to adopt child if £1 a week guaranteed, or would take Indian children.

73 Royal Parade, Eastbourne. Principal, Sister Collins. Personally known to member of The Hospital staff. All cases.

